# The Reformed Public House

**Published:** 1924-02

**Authors:** John A. Pace

**Affiliations:** Temple, E.C. 4


SIR,—Messrs. Dering and Grubb both appear to be arguing in favour of State purchase and control of the liquor trade and yet trying to cut one another's throats at the same time (metaphorically speaking). Mr. Dering advocates houses on the lines of the People's Refreshment House Association plans and states that the present system is only a money-making concern in the interests of shareholders, forgetting that a profit is also aimed at by the Association. He also, apparently, forgets that, excellent as are many of the houses of that Association, there are also equally good, if not in some cases even better and more up to date, privately-owned houses, some of which, indeed, are far superior to the much-vaunted State-controlled houses.

Mr. Grubb, equally emphatic on State control, will have nothing to do with limited liability companies and yet suggests "direct disinterested management," which is also carried on by just such limited liability companies. As to the amount of "disinterestedness" under those conditions, the manager gets his salary for holding a position that otherwise would not be open to him if there was no demand for a house that could supply alcoholic beverages as well as food. Take away that facility and the demand for food alone would be satisfied by the abundant teashops, etc., in the immediate vicinity—such disinterested management!

Temple, E.C. 4. JOHN A. PACE.

